# Supplementary material for: Investigating the Antibacterial Activity and Safety of Zinc Oxide Nanoparticles versus a Commercial Alcohol-Based Hand-Sanitizer: Can Zinc Oxide Nanoparticles Be Useful for Hand Sanitation?
Source: Antibiotics (Basel). 2022 Nov 11;11(11):1606. doi: 10.3390/antibiotics11111606 (PMC9686634; doi:10.3390/antibiotics11111606)
Supplement: Supplementary file 1 [file antibiotics-11-01606-s001.zip › antibiotics-1987398-supplementary.pdf]

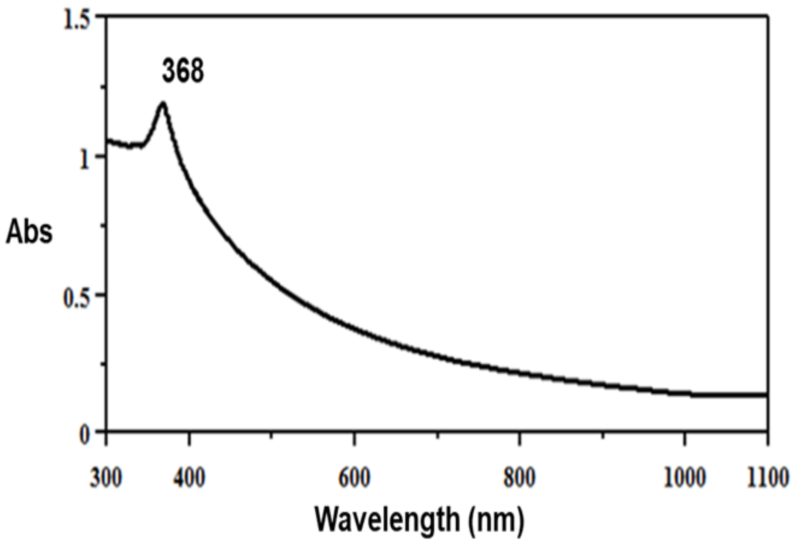

Figure S1. UV-visible absorbance spectroscopy of ZnO-NPs.

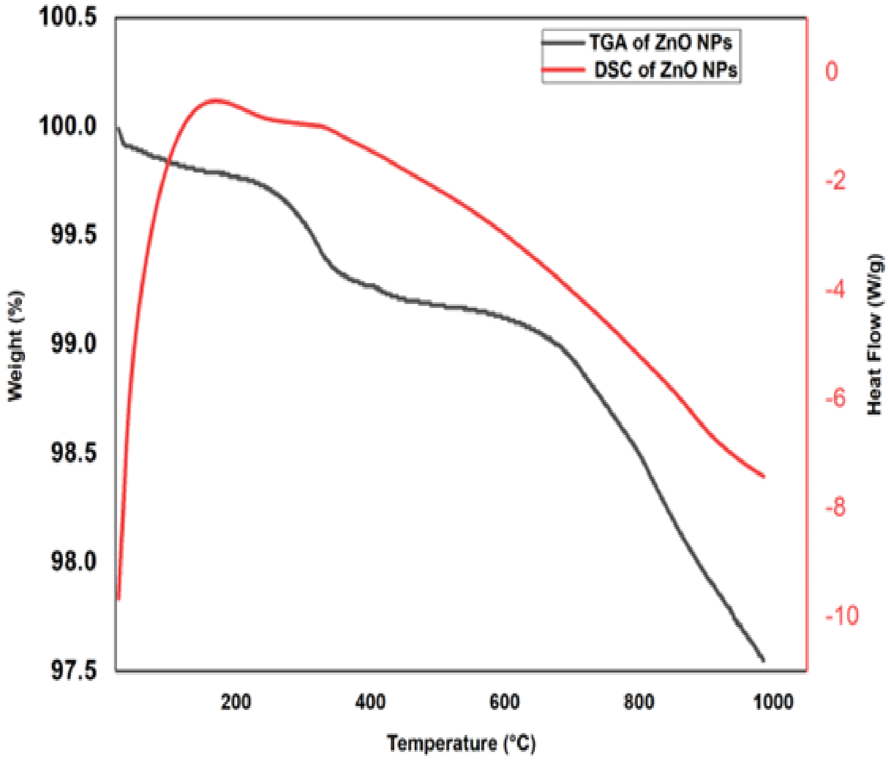

Figure S2. DSC-TGA thermogram of ZnO-NPs.

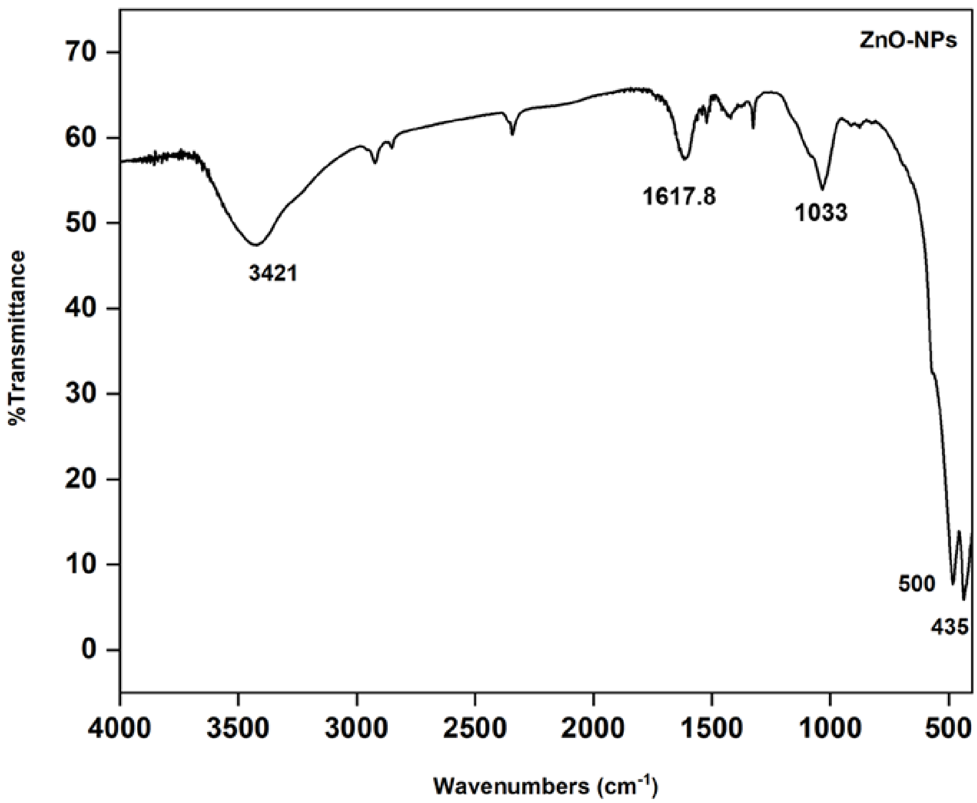

Figure S3. FTIR spectrum of synthesized ZnO-NPs.

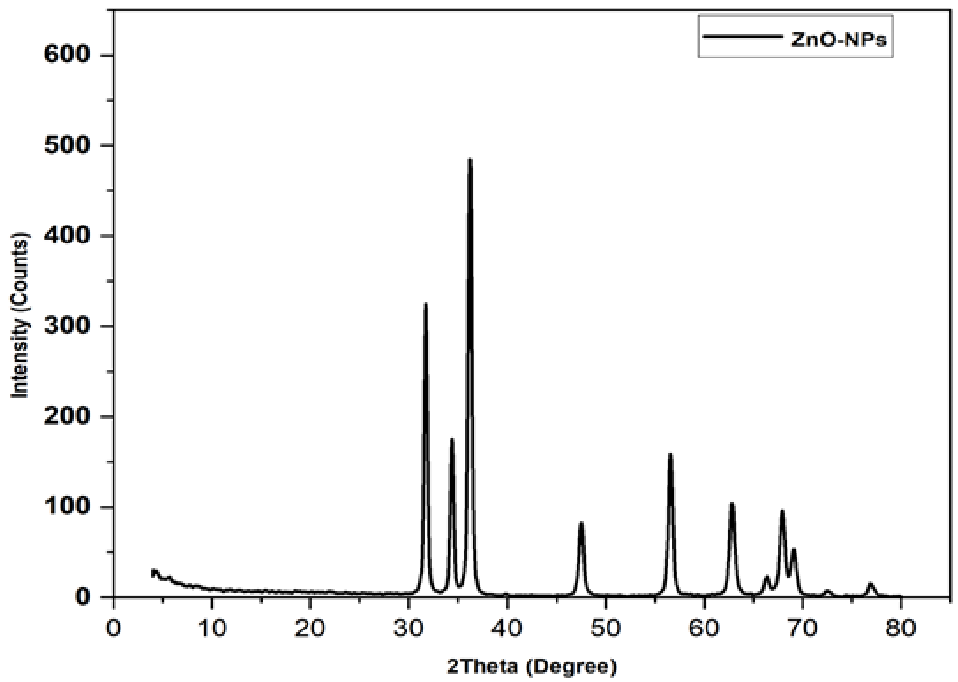

Figure S4. X-ray diffraction of synthesized ZnO-NPs.

Table S1: Stability of ZnO-NPs prepared under different Temperature and PVP concentration, results are average of three independent experiments, each involved a triplicate

PVP concentration- Day of measurement after preparation	*Preparation Condition		
	Particle Size (Dnm ± SD)	Polydispersity Index (PDI) ± SD	Zeta Potential (mV ± SD)
3% w/v – Day 0	361.7±2.08	0.302±0.008	-18.2±0.14
3% w/v – Day 1	378.7±2.25	0.323±0.03	-14.93±0.5
3% w/v – Day 2	445.3±13.5	0.37±0.043	-11.6±0.11
3% w/v – Day 3	587.4±46	0.37±0.019	-7.4±0.25
10% w/v - Day 0	349.6±11	0.311±0.006	-10.6±0.2
10% w/v - Day 1	358±14.3	0.345±0.045	-13.17±0.153
10% w/v - Day 2	503±6	0.29±0.025	-13.6±0.47
10% w/v - Day 3	501.6±4.56	0.288±0.025	-13.63±0.47

*Preparation Condit3ion; pH=8, Temperature = 70°C, RPM = 1500

**Table S2: Antimicrobial susceptibility profile of *S. aureus* ATCC 12600**	
**Antibiotics**	**Susceptibility**
Gentamicin (GN)	S
Tobramycin (TOB)	S
Amikacin (AK)	S
Oxacillin (OXA)	S
Amoxicillin/clavulanate (AMC)	S
Piperacillin (PRL)	S
Cefotaxime (CTX)	R
Ceftriaxone (CRO)	S
Imipenem (IMP)	R
Meropenem (MEM)	R
Ciprofloxacin (CIP)	S
Levofloxacin (LEV)	S
Vancomycin (VA)	S

**Table S3: Antimicrobial susceptibility profile of MRSA ATCC 25923**	
**Antibiotics**	**Susceptibility**
Gentamicin (GN)	R
Tobramycin (TOB)	R
Amikacin (AK)	R
Oxacillin (OXA)	R
Amoxicillin/clavulanate (AMC)	R
Piperacillin (PRL)	S
Cefotaxime (CTX)	R
Ceftriaxone (CRO)	R
Cefoxitin (FOX)	S
Imipenem (IMP)	R
Meropenem (MEM)	R
Ciprofloxacin (CIP)	R
Levofloxacin (LEV)	S
Vancomycin (VA)	S

**Table S4: Antimicrobial susceptibility profile of *K. pneumoniae* ATCC 10031**	
**Antibiotics**	**Susceptibility**
Gentamicin (GN)	S
Tobramycin (TOB)	S
Amikacin (AK)	S
Amoxicillin/clavulanate (AMC)	R
Piperacillin (PRL)	S
Cefotaxime (CTX)	R
Ceftriaxone (CRO)	R
Imipenem (IMP)	S
Meropenem (MEM)	S
Ciprofloxacin (CIP)	R
Levofloxacin (LEV)	S

**Table S5: Antimicrobial susceptibility profile of *Acinetobacter baumannii* ATCC 15308**	
**Antibiotics**	**Susceptibility**
Gentamicin (GN)	R
Tobramycin (TOB)	S
Amikacin (AK)	S
Amoxicillin/clavulanate (AMC)	R
Piperacillin (PRL)	S
Cefotaxime (CTX)	R
Ceftriaxone (CRO)	S
Imipenem (IMP)	R
Meropenem (MEM)	R
Ciprofloxacin (CIP)	R
Levofloxacin (LEV)	S
